# A Mouse Model of Neurodegeneration Induced by Blade Penetrating Stab Wound to the Hippocampus

**DOI:** 10.3390/biology11091365

**Published:** 2022-09-18

**Authors:** Bao-Dong He, Chang-Mei Liu, Zhao-Qian Teng

**Affiliations:** 1 State Key Laboratory of Stem Cell and Reproductive Biology, Institute of Zoology, Chinese Academy of Sciences, Beijing 100101, China; 2 Savaid Medical School, University of Chinese Academy of Sciences, Beijing 100049, China; 3 Beijing Institute for Stem Cell and Regenerative Medicine, Beijing 100101, China; 4 China Institute for Stem Cell and Regeneration, Chinese Academy of Sciences, Beijing 100101, China

**Keywords:** mouse model, neurodegeneration, learning and memory deficits, stab wound, traumatic brain injury, hippocampus

## Abstract

**Simple Summary:**

To date, various animal models of traumatic brain injury (TBI) have been developed to investigate the cellular and molecular networks underlying the pathogenesis of neurodegeneration, and to examine the safeness and therapeutic efficacy of drugs. However, the commonly used animal TBI models usually have the weaknesses of low reproducibility and high mortality rates. In the present study, we created a mouse model of cognitive deficits induced by a blade penetrating stab wound to the hippocampus (HBSI). This model will contribute a better understanding of neurodegeneration and accelerate the translation of preclinical research to clinical applications.

**Abstract:**

Traumatic brain injury (TBI) is closely associated with the later development of neurodegenerative and psychiatric diseases which are still incurable. Although various animal TBI models have been generated, they usually have weaknesses in standardization, survivability and/or reproducibility. In the present study, we investigated whether applying a blade penetrating stab wound to the hippocampus would create an animal model of cognitive deficits. Open-field, Morris water maze and Barnes maze tests were used to evaluate the animal behaviors. The immunofluorescence staining of NeuN, GFAP, IBA1, and TUNEL was conducted to analyze the changes in neurons, astrocytes, and microglia, as well as cell death. Mice with a hippocampal blade stab injury (HBSI) displayed the activation of microglia and astrocytes, inflammation, neuronal apoptosis, and deficits in spatial learning and memory. These findings suggest that HBSI is an easy approach to generate a reliable in vivo model of TBI to capture hemorrhage, neuroinflammation, reactive gliosis, and neural death, as well as cognitive deficits observed in human patients.

## 1. Introduction

Neurodegenerative disorders are still incurable and impose huge medical and economic burdens worldwide. Mutations in some causative genes (such as *PARKIN*, *PINK1*, *APOE*, *APP*, *PSEN1/2* and *TREM2*) are responsible for the disease phenotype and underlying pathogenesis [1]. However, many patients with neurodegenerative disorders have no clear genetic reasons, and the interactions of non-genetic and genetic factors are likely required for the occurrence of neurodegenerative disorders [2,3].

Traumatic brain injury (TBI) is a common cause for developing neurodegenerative diseases and psychiatric disorders [4,5]. TBI occurs as a result of a bump, blow, blast, crush or penetrating injury to the head which triggers a series of biochemical events that lead to neurodegeneration and cognitive impairment. Unfortunately, at present there is still a lack of drugs with clinical efficacy for the treatment of TBI and neurodegeneration [6]. To seek fundamental knowledge about the injured brain and use that knowledge to develop effective therapeutics for TBI, various animal models including fluid percussion, controlled cortical impact, ballistic-like penetrating, blast, and weight-drop injury models have been developed to replicate the various aspects of human TBI [7]. Although these animal models have the strengths of injury biomechanics similar to those seen in human TBI and/or allow the fine-tuning of injury severity, they have the weaknesses of a lack of standardization, high mortality rates, and low reproducibility as well [7].

The hippocampus is a key region of the brain that is involved primarily with learning and memory. The hippocampus is more prone to injury due to various reasons, such as having a complex structure containing dentate gyrus and Cornu ammonis (which is divided into CA1, CA2, CA3, and CA4) [8], a delicate balance between excitatory and inhibitory neurotransmission in the hippocampal neurons [9], and a high energy demand [10,11]. In addition, a decline in, or absence of, adult neurogenesis during aging makes human hippocampi more vulnerable to damage [12,13,14]. To date, simple and phenotypically stable animal models are much needed to investigate the pathogenetic mechanisms of hippocampal degeneration and to examine the therapeutic efficacy of drugs.

Here, we examined whether a hippocampal stab wound could induce neural degeneration and cognitive deficits in mice, and evaluated the reproducibility of this type of TBI model.

## 2. Materials and Methods

### 2.1. Animals

All mice used in the present study were of a C57BL/6J genetic background and bought from SPF (Beijing, China) Biotechnology Co., Ltd. The mice were kept on a 12 h:12 h light–dark cycle, with controlled temperature and humidity, and were housed in home cages with access to food and water.

### 2.2. Blade Penetrating Stab Wound

**Figure 1:**
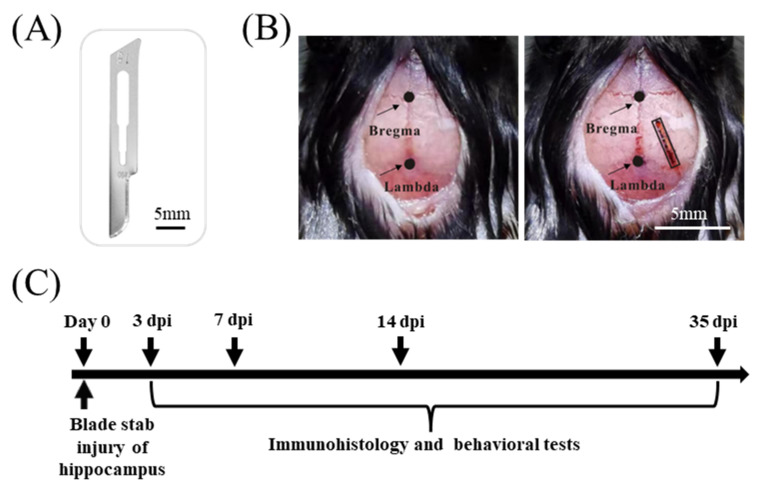
Hippocampal lesion by blade penetrating stab wound. (**A**) No. 15 scalpel blade used for penetrating stab wound to the hippocampus. Scale bar, 5 mm. (**B**) Hippocampus position and blade stab lesion. Stereotactic coordinates from bregma for marking a rectangular frame to determine the right hippocampus: upper-right corner, AP = 1.4 mm, ML = 1.4 mm; bottom-right corner, AP = 4.2 mm, ML = 2.1 mm; upper-left corner, AP = 1.6 mm, ML = 0.9 mm; bottom-left corner, AP = 4.5 mm, ML = 2.6 mm. Following the removal of a rectangular piece of skull using the No. 12 scalpel blade, hippocampus is wounded by inserting the tip of the No. 15 scalpel blade 3mm deep into the exposed brain region. Scale bar, 5 mm. (**C**) Experimental design of the study. Day 0 refers to the day of hippocampal blade stab injury (HBSI) or sham procedure. Eight-week-old male mice are chosen to subject to HBSI. After HBSI, behavioral assays of open-field, Morris water maze and Barnes maze tests are conducted to assess learning and memory dysfunction at 14, 15 and 35 days post-injury (dpi), respectively. Immunohistochemical staining is performed to detect neural degeneration (at 3 dpi) and gliosis (at 7 and 35dpi).

Male mice at 8 weeks of age were anesthetized with 200 mg/kg Avertin (T48402, Sigma-Aldrich, St. Louis, MO, USA). Mice were positioned in the KOPF stereotaxic apparatus. Midline skin incisions were made to expose the skull. A cranial window (1 mm in width, 4 mm in length) was opened by a No. 12 scalpel blade on the right side of the skull, and a hippocampal lesion was made by inserting a sterile scalpel blade #15 (10015-00, Fine Science Tools; Figure 1A) which was held on the KOPF stereotaxic apparatus with the coordinates of the right hippocampus as follows (from bregma) [15]: upper-right corner, AP (anterior-posterior) = 1.4 mm, ML (mediolateral) = 1.4 mm; bottom-right corner, AP = 4.2 mm, ML = 2.1 mm; upper-left corner, AP = 1.6 mm, ML = 0.9 mm; bottom-left corner, AP = 4.5 mm, ML = 2.6 mm (Figure 1B). The blade was allowed to stay in the hippocampus for 1 min before slowly being removed in order to avoid blood backflow along the stab tract. The skin surrounding the wound was cleaned with a sterile swab and closed with an absorbable surgical suture. Finally, the mice were returned to their home cages once they were freely moving around.

### 2.3. Behavioral Tests

Mice were transported to the testing room 24h before the behavioral tests for acclimation. All behavioral assays were performed within a given time window (Figure 1C) during the light phase between 07:00 and 19:00, as previously described [16,17,18]. The videos were recorded and analyzed using the software Smart V3.3.03 (Pan Lab, Harvard Apparatus, Holliston, MA, USA).

**Open-field test**: The open-field test was conducted in a plywood box (72 cm long × 72 cm wide × 36 cm tall). An 18 × 18 cm square was drawn in the center of the open field. The mice were randomly placed in a corner of the box and allowed to freely explore the box for 5 min. The total distance moved in the box and the number of center entries were calculated.

**Barnes maze test**: The Barnes maze is a circular platform (120 cm in diameter) with 20 evenly spaced holes with diameters of 5 cm and 2 cm away from the edge. There is only one hole leading to an escape box. Paper pieces with triangle, circle, and cross shapes were pasted on the walls as environmental cues for the mice. The test procedure was composed of three phases: habituation (day 1), training (days 2–3) and probe (day 5). On the habituation day, mice were placed in the center of the maze and were given 2 min to explore the hiding box. For mice that did not find the escape cage by the end of 2 min, they were gently guided to the hiding box and allowed to stay in it for 1 min. In the training phase, the mice were allowed to begin searching for the hiding box from the center of the maze for 2 min. If the mice failed to find the hiding box after 2 min, they were manually guided to the hiding box. Three trials on training day 1 and two trials on training day 2 were conducted for every mouse. Finally, a 2 min probe trial was performed 48 h following the last day of training. Measures of primary latency and the number of visits to the escape hole were recorded.

**Water maze test**: A 120 cm diameter circular tank was filled with opaque water (21 ± 1 °C) using nontoxic white paint. At the center of a given quadrant of the water tank, a 13 cm diameter platform was hidden 1.5 cm beneath the surface of the water. The spatial acquisition phase consisted of four training trials per day for 4 successive days. For each trial, the mice were gently released from the pool wall of a randomly selected quadrant and allowed to swim and locate the platform. If the mice did not find the platform within the 60 s trial period, they were manually guided onto the platform and allowed to stay on it for 30 s. By removing the platform, proof trials were performed 24 h after the completion of the last spatial acquisition. The swimming speeds, time spent and tracks were recorded.

### 2.4. Immunofluorescence Assay

An immunofluorescence assay was performed according to published protocols [17,19]. The mice were anesthetized with 200 mg/kg Avertin and transcardially perfused with cold 1× PBS followed by 4% paraformaldehyde in a phosphate buffer (PFA). Brain tissue was dissected out and fixed in 4% PFA overnight at 4 °C. Fixed brains were stored in a 30% sucrose solution at 4 °C until they sank. The brains were sectioned into 40 μm thick serial sections using the Leica Sliding Microtome (Wetzlar, Germany) (SM2010 R). The brain sections were washed in 1× PBS three times for 30 min, then treated in a blocking solution (2% BSA, 0.3% Triton X-100) at room temperature for 1 h. After incubating with primary antibodies in the blocking solution at 4 °C overnight, the brain sections were washed with 1× PBS three times for 30 min and then incubated with DAPI, and the secondary antibodies were conjugated to Alexa Fluor 488 or 647 (1:500) at room temperature for 2 h. Finally, the brain sections were mounted on slides using an adhesion anti-fade medium and stored in the dark. The primary antibodies included anti-IBA1(1:500, NB100-1028, NOVUS, Murfreesboro, TN, USA), anti-GFAP (1:500, 16825-1-AP, Proteintech, Rosemont, IL, USA), and anti-NeuN (1:500, MAB377, Millipore, St. Louis, MO, USA).

### 2.5. Quantitative Real-Time Polymerase Chain Reaction (qRT-PCR)

The total RNA of each hippocampal tissue was extracted using a TRIzol reagent (Invitrogen, Waltham, MA, USA) according to the manufacturer’s manual. The RNA concentration was measured using ultraviolet spectrophotometry, and only the samples with an A260/A280 ratio of >2.0 were used for further analysis. For cDNA synthesis, 1 μg of total RNA was transcribed using the TransScript One-Step gDNA Removal and cDNA Synthesis SuperMix (TRANS) kit. A qRT-PCR was performed using the SYBR^®^ Green PCR Master Mix (Yeasen, Shangai, China). The primers were subjected to 45 cycles of amplification at 95 °C for 10 s and 60 °C for 30 min. β-actin was used as an internal control and the gene expression levels were normalized to that of GAPDH using the 2 − ΔΔCt (threshold cycle) method. The primers we used for qRT-PCR were as follows: TNF-α (forward 5′-CTCTTCTGCCTGCTGCACTTTG-3′, reverse 5′-ATGGGCTACAGGCTTGTCACTC-3′), IL1-β: (forward 5′-CCACAGACCTTCCAGGAGAATG-3′, reverse 5′-GTGCAGTTCAGTGATCGTACAGG-3′), iNOS (forward 5′-GGCAGCCTGTGAGACCTTTG-3′, reverse 5′-GCATTGGAAGTGAAGCGTTTC-3′), β-actin (forward 5′- GCCACCAGTTCGCCATGGAT-3′, reverse 5′- CATCACACCCTGGTGCCTAG-3′).

### 2.6. Statistical Analysis

Statistical data analyses were conducted using GraphPad Prism V9.0.0 (GraphPad Software, San Diego, CA, USA). Statistical significance is defined at the 5% level by either AVONA with post hoc tests or an unpaired Student’s two-tailed *t*-test. Data are presented as mean ± SEM.

## 3. Results

### 3.1. HBSI Triggers an Upregulation of Proinflammatory Cytokines in the Hippocampus

**Figure 2:**
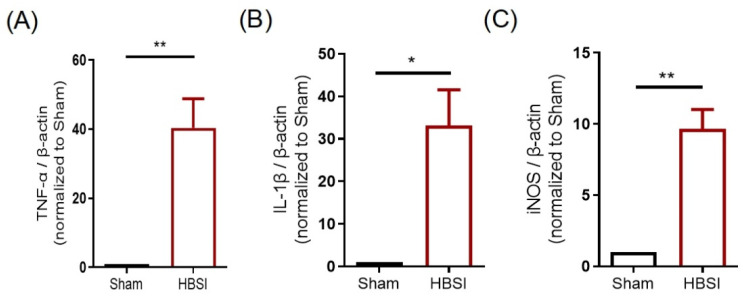
HBSI triggers an upregulation of proinflammatory cytokines in the hippocampus at 3 days post-injury. (**A**) TNF-α, (**B**) IL-1β, and (**C**) iNOS mRNA expression levels were significantly increased in the hippocampi at 3 dpi by qRT-PCR. β-Actin was used as an internal control. Eight male mice were randomly chosen from 4 litters, with 2 males from each litter. In each litter, one mouse underwent HBSI and the other mouse underwent a sham treatment. To quantify relative mRNA levels, each value of the HBSI male was divided by the value of his sham littermate to normalize the data. Data are presented as means ± SEM. * *p* < 0.05, ** *p* < 0.01.

As inflammation plays a critical role in the pathophysiology of traumatic brain injury [20,21], we used qRT-qPCR to detect the expression levels of several main proinflammatory cytokines in the hippocampus at 3 days after HBSI. We found that the mRNA expression levels of TNF-α (Figure 2A), IL-1β (Figure 2B), and iNOS (Figure 2C) were dramatically elevated after HBSI, which suggested that HBSI could trigger an acute inflammation in the early stages of injury.

### 3.2. HBSI Results in Augmented Gliosis in the Hippocampus

**Figure 3:**
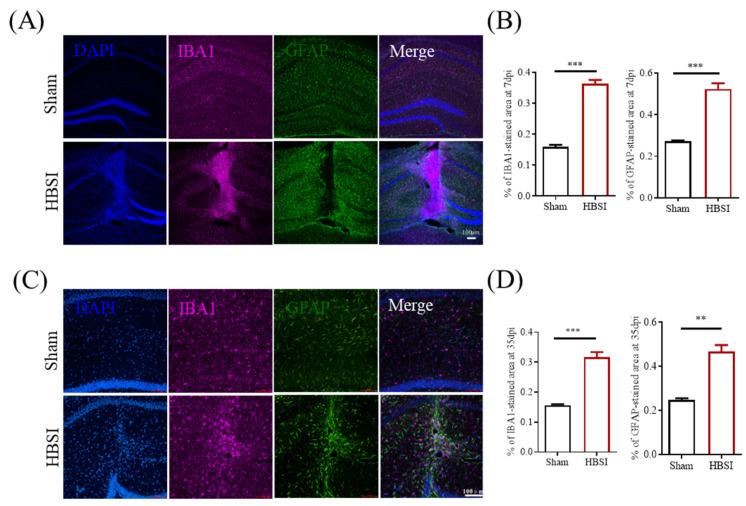
Reactive astrogliosis and microgliosis in hippocampus following stab injury. (**A**) Representative images and (**B**) quantification of GFAP and IBA1 immunostainings demonstrated that the injured hippocampus has dramatically augmented microgliogenesis and astrogliogenesis compared to the contralateral uninjured hippocampus at 7 dpi. *n* = 4 mice per group. (**C**) Representative images and (**D**) quantification of IBA1 and GFAP immunostainings demonstrate that the injured hippocampus has significantly elevated microgliogenesis and astrogliogenesis compared to the uninjured hippocampus at 35 dpi. Scale bars, 100 µm. *n* = 4 mice per group. Data are shown as means ± SEM. ** *p* < 0.01, *** *p* < 0.001.

Following TBI, resident astrocytes and microglia are the main cell types to initiate an inflammatory response, and the activations of microglia and astrocytes are believed to be biomarkers for an accurate diagnosis of TBI [22,23,24]. On day 7 after HBSI, IBA+ microglia were confined to the lesion core, surrounded by the reactive astrocytes that separate the glial scar from the surrounding healthy tissue (Figure 3A). In the contralateral uninjured hippocampus, IBA+ microglia were small and few in number, and were distributed evenly across the entire tissue (Figure 3A). A quantification of GFAP+ and IBA1+ cells demonstrated that the injured hippocampi had dramatically augmented microgliogenesis and astrogliogenesis compared to the uninjured hippocampi at 7 dpi (Figure 3B). On day 35 after HBSI, although the glial scar was slightly loosened (Figure 3C), the numbers of GFAP+ and IBA1+ cells were still significantly higher in the HBSI hippocampi than those in the uninjured hippocampi (Figure 3D).

### 3.3. HBSI Leads to Neuronal Apoptosis in the Hippocampus

**Figure 4:**
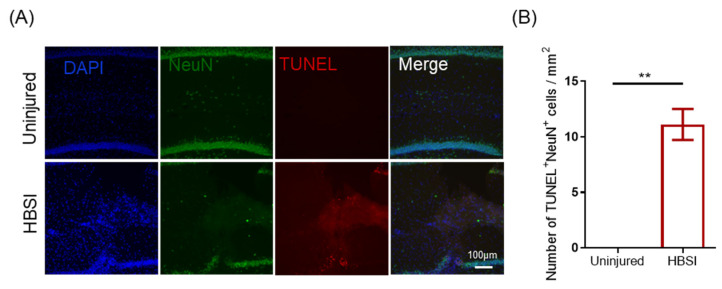
Neuronal apoptosis occurs after HBSI. (**A**) Representative images and (**B**) quantification of NeuN and TUNEL immunostainings showed that mice with HBSI had more neurons undergoing apoptosis in the hippocampus compared to that in uninjured hippocampus at 3 dpi. Scale bar, 100 µm. *n* = 4 mice per group. Data are shown as means ± SEM. ** *p* < 0.01.

In order to determine whether neuronal apoptosis occurs after HBSI, we performed double immunofluorescent staining of the neuronal nuclei (NeuN) and terminal deoxynucleotidyl transferase-dUTP nick end labeling (TUNEL) to quantify TUNEL+ and NeuN+ cells in both the sham and HBSI hippocampi at 7 dpi. As we expected, more TUNEL+NeuN+ cells were observed in the HBSI hippocampal tissues adjacent to the wound track than that of the contralateral uninjured hippocampi (Figure 4A,B). Therefore, HBSI led to hippocampal neurons undergoing degeneration after the injury.

### 3.4. Mice with HBSI Exhibit Deficits in Learning and Memory

**Figure 5:**
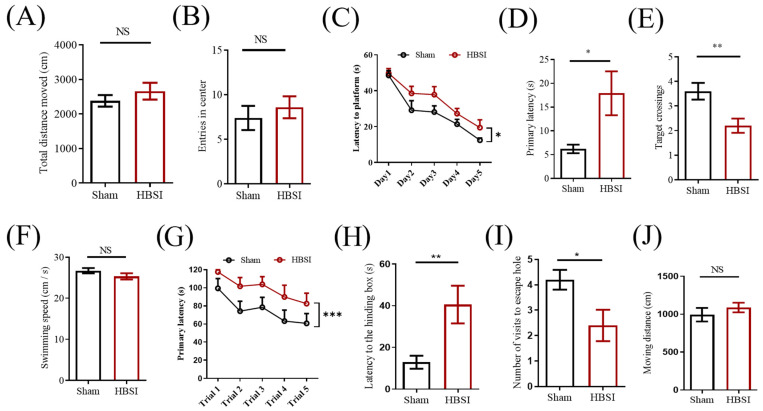
Mice with HBSI exhibited deficits in learning and memory. (**A**,**B**) Open-field test was performed at 14 days after HBSI. There were no significant differences in travel distance (**A**) and entries in center (**B**) between the sham and HBSI groups. *n* = 10 mice per group. (**C**–**F**) Morris water maze test was started to evaluate the spatial learning and memory of mice at 14 days after HBSI. During the training phase, both sham and HBSI groups showed improved latency to search for the platform, but mice with HBSI exhibited a significant delay in finding the platform compared to sham group (**C**). In the probe phase, mice with HBSI exhibited a dramatically longer latency to locate the target (**D**) and fewer target crossings (**E**) compared with the sham group. However, there was no significant difference in swimming speed between the sham and HBSI groups (**F**) *n* = 10 mice per group. (**G**–**J**) Barnes maze test was conducted at 35 days post-HBSI. During the training phase, mice with HBSI took a longer time to enter into the hiding box compared to sham control (**G**). In the probe test, injured mice exhibited a significantly longer latency to locate the hiding box (**H**) and had fewer visits to escape hole (**I**) compared to sham control. There was no significant difference in total moving distance between the sham and HBSI groups in the Barnes maze test (**J**) *n* = 10 mice per group. Data are presented as means ± SEM. NS, non-significant; * *p* < 0.05, ** *p* < 0.01.

Next, we sought to determine whether unilateral HBSI could impair learning and memory. Firstly, we performed an open-field test to measure locomotor activity and anxiety-like behavior in the mice at 14 dpi. There was no obvious difference in the total moving distance during the 5 min period (Figure 5A), suggesting similar locomotor activities between the sham and HBSI mice. Meanwhile, the injured mice entered the center zone with the same times as the control mice (Figure 5B), indicating that they had no obvious anxiety-like behavior.

The Morris water maze test was then conducted at 15 dpi to evaluate the spatial learning and memory in the HBSI mice. During the training phase, although mice in both the sham and HBSI groups showed improved latency in finding the platform, mice with HBSI spent a longer time to locate the platform than the sham mice (Figure 5C). In the subsequent probe test, mice with HBSI exhibited deficits in learning and memory with a longer time to find the platform (Figure 5D) and a smaller number of target crossings (Figure 5E) compared to the sham controls. On the other hand, there was no significant difference in swimming speed between the sham and HBSI groups (Figure 5F), suggesting that the different latency to locate the platform and the different target crossings were due to different learning and memory processes, but not to differences in the speed of swimming between the sham and HBSI groups.

To further validate the impaired cognitive function of the HBSI mice, we performed a Barnes maze test at 35 dpi. During the training phase, HBSI mice did take a longer time to enter into the hiding box compared to the sham controls (Figure 5G). Consistently, in the probe test, HBSI mice exhibited a significantly longer latency to find the escape box (Figure 5H), and had fewer visits to the escape hole (Figure 5I) compared to the sham controls. The total moving distance was not significantly different between the sham and HBSI groups (Figure 5J). These behavioral data strongly support that unilateral HBSI could efficiently impair learning and memory in mice.

## 4. Discussion

TBI is a complex disease process which displays structural damage and functional deficits that are caused by both primary and secondary injury mechanisms [25,26]. The primary injury is directly caused by a mechanical disruption of brain tissue that occurs at the time of exposure to a surface contusion, blast, hemorrhage or axonal shearing [27,28]. The secondary injury evolves from the primary injury that is created by a cascade of neuroinflammation, neurodegeneration, and atrophy [29,30]. However, currently there is no one animal model which is able to recapitulate all aspects of the clinical pathogenesis observed in human TBI [7]. In this study, we observed that the unilateral HBSI had no obvious effects on gliosis nor neuronal apoptosis in the contralateral hippocampus (Supplementary Figure S1), suggesting that unilateral HBSI belongs to mainly focal, not diffuse, types of injuries. However, we found that HBSI could capture hemorrhage, neuroinflammation, and neuronal apoptosis in the injured hippocampus. As a result, mice with unilateral HBSI exhibited deficits in spatial learning and memory. Moreover, all HBSI mice survived as long as the sham controls. Compared with other TBI models which often require sophisticated measures using electrical (e.g., Leica Impact One Controlled Impact Device) or mechanical equipment (e.g., weight-drop devices), our HBSI model is generated with an easy procedure by using ordinary surgery tools. Thus, the HBSI model should be very useful to assist researchers in investigating neurodegenerative mechanisms, and in developing new strategies to treat neurotrauma and cognitive disorders.

Similar to other animal models, the mouse model of HBSI also has several limitations. Firstly, the experimenter needs to use the stereotaxic apparatus to practice many times in order to accurately damage the mouse hippocampus, due to its relatively small size. We speculate that HBSI should be more easily applied in generating models of TBI based on large animals (such as rats, pigs and monkeys). Secondly, the surgical method of HBSI not only damages the hippocampus, but also the cerebral cortex adjacent to it. Since the cerebral cortex is involved in many higher-level processes such as reasoning, mood, language, learning and memory [31], caution should be taken when applying the HBSI model to interpret any changes in animal behaviors. Thirdly, extensive differences between human and mouse brains in anatomy, cell types, gene expression and morphology have been important factors underlying current failures of mouse models for translation into human patients [32,33]. With the advancement of human brain organoid technology [34,35], applying both the human brain organoid and primate models may be a better approach towards an understanding and treatment of neurotrauma and neurodegeneration.

## 5. Conclusions

The main finding of the present study is that a blade stab injury of a unilateral hippocampus is a reliable method to generate a homogenous mouse model of TBI. HBSI can efficiently induce inflammation, reactive gliosis, and neural death, as well as impairment in learning and memory. We speculate that the HBSI method should also be applicable in generating primate and human brain organoid models of TBI to facilitate understanding and developing new treatments of neurotrauma and neurodegeneration.

## Data Availability

All data generated or analyzed in this study are available on request from the corresponding authors.

## Supplementary Materials

The following supporting information can be downloaded at: https://www.mdpi.com/article/10.3390/biology11091365/s1, Figure S1: The contralateral uninjured hippocampi in unilateral HBSI mice have similar effects on gliosis and neuronal apoptosis as that in the sham group. (A) Representative images and quantification of IBA1 and GFAP immunostainings demonstrated that the uninjured hippocampus has no significant difference in microgliogenesis and astrogliogenesis compared to the untreated hippocampus at 7 dpi. (B) Representative images and quantification of IBA1 and GFAP immunostainings demonstrate that the uninjured hippocampus has no significant difference in microgliogenesis and astrogliogenesis compared to the untreated hippocampus at 35 dpi. (C) Representative images and quantification of TUNEL and NeuN immunostainings demonstrate that the uninjured hippocampus has no significant difference in neural apoptosis compared to the untreated hippocampus at 3 dpi. Scale bars, 100 µm. n = 4 mice per group. Data are presented as means ± SEM. NS, non-significant.
