# Supplementary material for: A Mouse Model of Neurodegeneration Induced by Blade Penetrating Stab Wound to the Hippocampus
Source: Biology (Basel). 2022 Sep 18;11(9):1365. doi: 10.3390/biology11091365 (PMC9495452; doi:10.3390/biology11091365)
Supplement: Supplementary file 1 [file biology-11-01365-s001.zip › biology-1886719-supplementary.pdf]

biology

MDPI

Supplementary Materials

A Mouse Model of Neurodegeneration Induced by Blade Penetrating Stab Wound to the Hippocampus

Bao-Dong He, Chang-Mei Liu and Zhao-Qian Teng

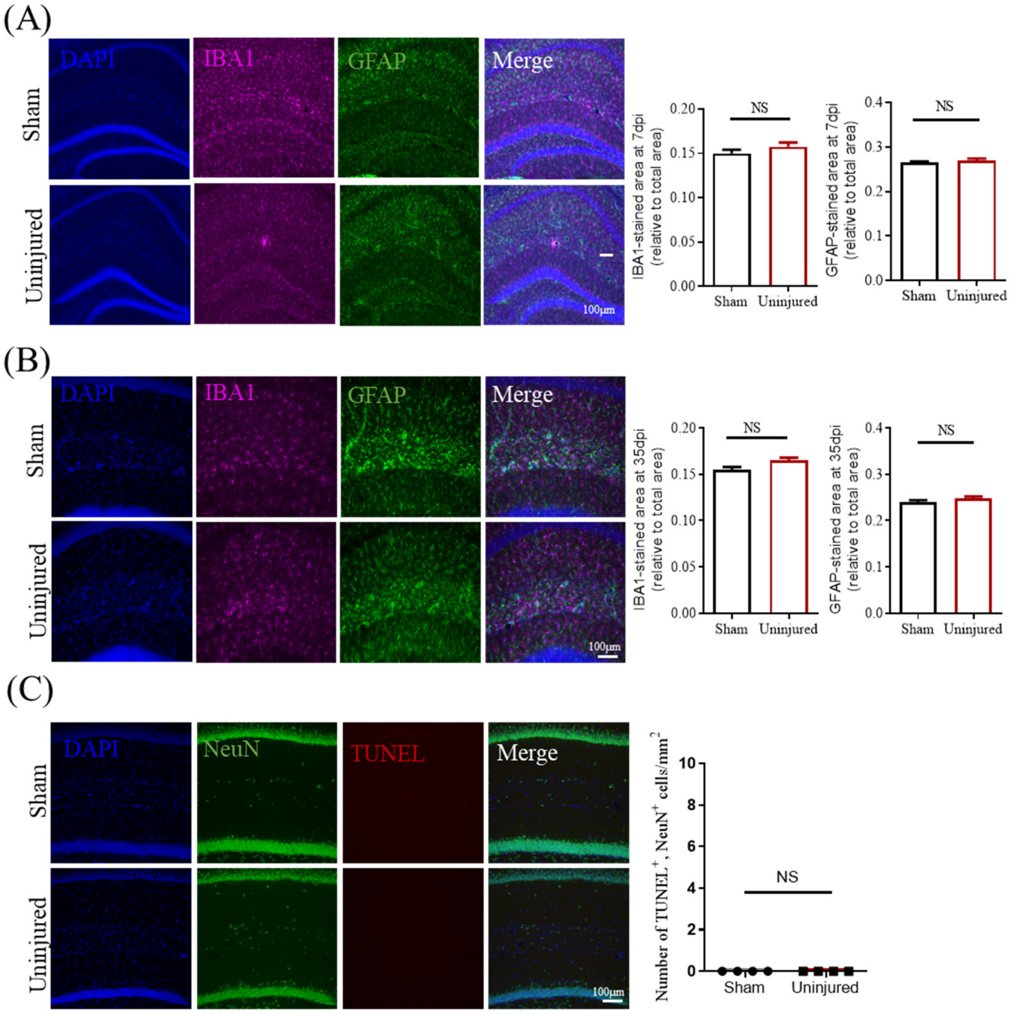

Figure S1. The contralateral uninjured hippocampi in unilateral HBSI mice have similar effects on gliosis and neuronal apoptosis as that in the sham group. (A) Representative images and quantification of IBA1 and GFAP immunostainings demonstrated that the uninjured hippocampus has no significant difference in microgliogenesis and astrogliogenesis compared to the untreated hippocampus at 7 dpi. (B) Representative images and quantification of IBA1 and GFAP immunostainings demonstrate that the uninjured hippocampus has no significant difference in microgliogenesis and astrogliogenesis compared to the untreated hippocampus at 35 dpi. (C) Representative images and quantification of TUNEL and NeuN immunostainings demonstrate that the uninjured hippocampus has no significant difference in neural apoptosis compared to the untreated hippocampus at 3 dpi. Scale bars, 100 µm. n = 4 mice per group. Data are presented as means ± SEM. NS, non-significant.
